# Scanning electron microscopy for blood micro-crystals in aortic stenosis patients

**DOI:** 10.1371/journal.pone.0202282

**Published:** 2018-08-23

**Authors:** David S. Wald, Elena Tsolaki, Jonathan P. Bestwick, Sergio Bertazzo

**Affiliations:** 1 Queen Mary University of London, Barts and The London School of Medicine and Dentistry, Wolfson Institute of Preventive Medicine, London, United Kingdom; 2 University College London, Department of Medical Physics & Biomedical Engineering, London, United Kingdom; Brigham and Women’s Hospital, Harvard Medical School, UNITED STATES

## Abstract

**Background:**

Micro-crystals of calcium phosphate have been detected on the aortic valve of patients with aortic stenosis using scanning electron microscopy. It is not known whether crystalisation is specific to heart valve tissue or a general blood-derived process.

**Methods:**

To this end we modified the method to determine whether calcium phosphate micro-crystals were present in the blood of patients with aortic stenosis. The method was first validated by adding synthetic calcium phosphate hydroxyapatite micro-crystals to healthy volunteer blood samples and determining the lower limit of detection. Then the method was used to examine the blood of 63 patients with echocardiographically confirmed aortic stenosis and 69 unaffected controls undergoing echocardiography for other reasons. Serum calcium and phosphate were measured and the calcium phosphate product compared in cases and controls.

**Results:**

In the validation study, synthetic hydroxyapatite micro-crystals were identified down to a lower concentration limit of 0.008mg/mL. In the experimental study no particles were identified in any patient, with or without aortic stenosis, even though serum calcium phosphate was higher in cases compared with controls 2.6mmol/L (2.58–2.77) versus 2.47mmol/L (2.36–2.57), p = 0.005 for the difference.

**Conclusion:**

The results of our study confirm a positive association between serum calcium phosphate and aortic stenosis, but indicate that the calcium phosphate particles found in valve tissue do not precipitate freely in the blood.

## Introduction

Aortic stenosis is a serious heart valve disorder affecting about 12% of people over the age of 75 [1]. It is caused by progressive deposition of calcified material on the aortic valve, leading to valve leaflet restriction and obstruction of blood flow from the heart. Death follows symptoms of cardiac insufficiency in the majority of cases, unless the aortic valve is surgically replaced. Progression from mild to severe disease occurs in most affected individuals, but the rate of progression varies widely; on average it is about 5 years [2]. There is no means of prevention.

Scanning electron microscopy has demonstrated micro-particles of calcified material on the aortic valve consisting of a highly crystalline form of calcium phosphate hydroxyapatite, distinct from bone mineral [3]. Whilst the number and density of micro-crystals increases with disease severity, the same fundamental particle is present at all stages of disease, from mild to severe aortic stenosis [3].

It is not known whether calcium phosphate micro-crystals are present in the blood of patients with aortic stenosis, and therefore whether precipitation is a general blood-derived process or specific to heart valve tissue. If present in the blood, their measurement would provide a potential means of screening for the disorder or identifying a group of patients at risk of rapid progression who may need earlier valve replacement.

We sought to apply the method for imaging calcium phosphate micro-crystals on aortic valve tissue to the blood of patients with and without aortic stenosis, to determine whether micro-crystals were present.

## Methods

Between July 2016 and January 2017, 63 consecutive patients with echocardiographically confirmed aortic stenosis were identified, from cardiology clinics and wards at Barts Heart Centre, London. Aortic stenosis was defined as thickened aortic valve leaflets with a peak trans-aortic velocity greater than or equal to 2m/s. We excluded patients with bicuspid aortic valve (5) or rheumatic valve disease (4). A further 69 patients, without aortic stenosis, who were undergoing echocardiography for other reasons, were invited to participate in the study as unaffected controls. Patients gave their written consent to participate in the study which was approved by the North West Preston Research Ethics Committee.

The method used for imaging calcification, by scanning electron microscopy in valvular tissue has been described [3]. The method was modified to detect micro-crystals in blood. A validation study was conducted on multiple blood samples from a healthy volunteer to which we added, across a range of concentrations, synthetic calcium phosphate hydroxyapatite micro-crystals (Sigma-Aldrich hydroxyapatite nanopowder 693863-5G) to determine the lower limit of detection by scanning electron microscopy. We started with a concentration of 0.5mg/mL, confirmed that the crystals were present and undertook sequential step-down dilutions (0.1mg/mL, 0.05mg/mL etc) until the crystals were no longer detectable and then stepped up in smaller concentration increments until they were once again detectable. The synthetic micro-crystals were similar in composition (95% hydroxyapatite), morphology (round), density (3.1g/mL) and size (200nm) to the particles found in aortic stenosis [3]. The samples were centrifuged at 1000g for 10 minutes, to separate red blood cells and hydroxyapatite micro-crystals from other blood components. Red blood cells mixed with micro-crystals were transferred to a new flask and centrifuged at 3600g. An aliquot of 5μL was extracted from the bottom of the flask, containing red blood cells plus micro-crystals, placed on a microscope slide and air-dried overnight. Once dried, the samples were coated with carbon using a Quorum K975X Carbon coater and imaged under a Hitachi S-3400N or Carl Zeiss Leo scanning electron microscope, using a voltage of 10kV. The backscattered electron mode was used to identify hydroxyapatite micro-crystals on the slide. This same method was applied to blood samples from patients affected with aortic stenosis and blood samples from the unaffected control group.

Peripheral blood samples were also collected for serum phosphate concentrations using the colorimetric reaction method with ammonium molybdate [4], and for total calcium, using indirect potentiometry [5]. Biochemical analyses were performed “blind”, that is without knowledge of which samples were cases or controls. Serum phosphate, serum calcium and the calculated calcium—phosphate product (an independent risk factor for aortic stenosis and target for treatment) [6] were compared using unpaired t-tests. Stata was used for all analyses.

## Results

Table 1 gives clinical and echocardiographic details of the patients with and without aortic stenosis in the study. There were no statistically significant differences between the groups apart from the expected differences in echocardiographic parameters reflecting the presence or absence of aortic stenosis in cases and controls respectively.

**Table 1:** Characteristics of individuals with and without aortic stenosis in the study.

	Cases (n = 63)	Controls (n = 69)	p-value
Age*	77 (69–84)	74 (65–81)	0.073
Male	35 (56)	45 (65)	0.288
Smoker	1 (2)	6 (9)	0.118
Medical history			
Myocardial infarction	12 (19)	16 (23)	0.357
Stroke	7 (11)	10 (14)	0.376
Renal failure	5 (8)	6 (9)	0.564
Diabetes	19 (30)	16 (23)	0.431
Echocardiographic parameters			
Peak trans-aortic velocity (m/s)*	3.6 (2.9–4.1)	1.3 (1.1–1.6)	<0.001
Peak trans-valvar gradient (mmHg)*	48 (32–68)	7.1 (5.4–11)	<0.001
Aortic Valve Area (cm^2^)*	0.8 (0.7–1.2)	2.4 (2.0–3.2)	<0.001

Values are number (%) except *median (IQR)

Table 2 gives the concentrations of serum calcium, serum phosphate and the calcium phosphate product (with 95% confidence internals), in cases and controls. For serum calcium these were, respectively, 2.37mmol/L (2.34–2.39) and 2.34mmol/L (2.32–2.37) (p = 0.154 for difference), for serum phosphate, 1.13mmol/L (1.09–1.17) and 1.05mmol/L (1.01–1.10) (p = 0.011 for difference) and for the calcium phosphate product, 2.67mmol/L (2.58–2.77) and 2.47mmol/L (2.36–2.57), p = 0.005 for the difference.

**Table 2:** Serum calcium, serum phosphate and the serum calcium phosphate product in 63 cases of aortic stenosis and 69 controls without aortic stenosis.

Serum level (mmol/L)	Mean (95% CI)		p-value
	Cases, n = 63	Controls, n = 69	
Calcium	2.37 (2.34–2.39)	2.34 (2.32–2.37)	0.154
Phosphate	1.13 (1.09–1.17)	1.05 (1.01–1.10)	0.011
Calcium × phosphate	2.67 (2.58–2.77)	2.47 (2.36–2.57)	0.005

In the validation study, synthetic hydroxyapatite micro-crystals were identified at a concentration of 0.5mg/mL and at serial step-down concentrations of 0.1mg/mL, 0.05mg/mL, 0.01mg/mL but not at 0.005mg/mL. Serial step-up concentrations from 0.005mg/mL identified a lower-limit of detection of 0.008mg/mL (S1 Fig).

**Fig 1:**
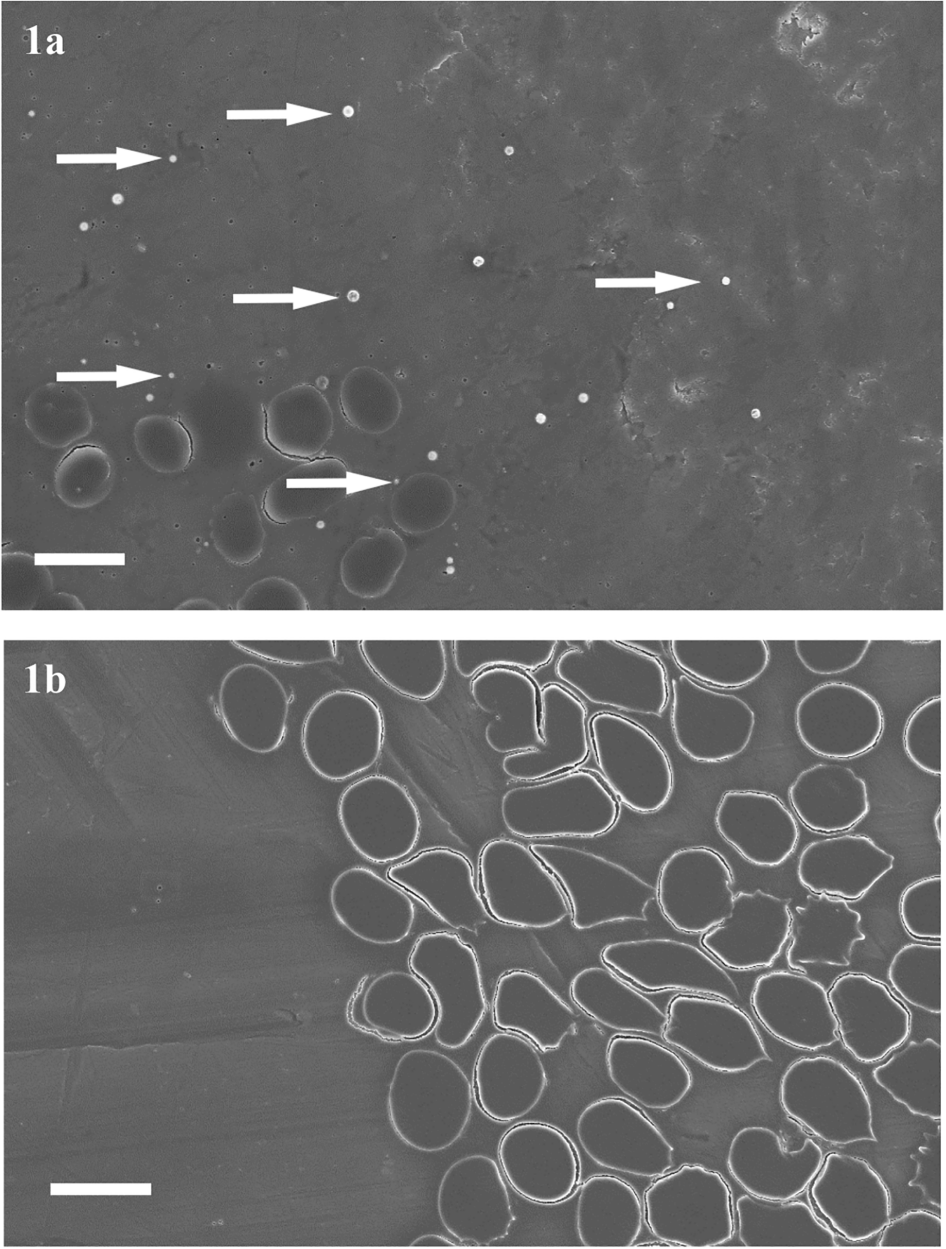
Scanning electron micrograph of red blood cells. (a) from healthy volunteer, mixed with synthetic calcium phosphate micro-crystals which are visible and indicated by arrows and (b) of red blood cells in a blood sample from a patient with aortic valve stenosis with no micro-crystals visible. Scale Bar = 10μm.

The images show the micro-crystals identified by arrows. Below 0.008mg/mL the crystals were not seen. The experiment was run twice and produced the same result on each occasion. In the case-control study no micro-crystals were identified in micrographs from any patient (affected or unaffected). An example from one patient with aortic stenosis is shown in Fig 1, which shows the red blood cells, but no micro-crystals and contrasts with a healthy volunteer sample to which synthetic micro-crystals were added.

## Discussion

The results show that scanning electron microscopy imaging methods used in the detection of calcium phosphate hydroxyapatite micro-crystals on aortic valve tissue can be modified to detect synthetic micro-crystals added to the blood of healthy volunteers. In spite of finding an increased concentration of calcium phosphate in the serum of patients with aortic stenosis, no circulating micro-crystals were identified.

Epidemiological studies show positive associations between the serum levels of calcium, phosphate, the calcium phosphate product and the presence and severity of calcific aortic valve stenosis [7–10]. Our results are consistent with these findings, showing a statistically significantly higher level of calcium phosphate in cases of aortic stenosis than controls without aortic stenosis. These associations, whilst positive, are insufficient in size, with odds ratios for aortic stenosis of about 1.5 per 0.1mg/dL increase in serum marker, to be worthwhile screening tests for the disorder [11], justifying the search for other screening markers.

It is known that increasing levels of calcium and phosphate, within the physiological range, induce calcification of valvular interstitial cells in vitro [12]. Serum is a metastable solution with respect to calcium phosphate precipitation. Once started, calcification proceeds rapidly in the presence of calcifiable templates such as collagen, elastin, and cell debris, in the same way as crystals grown from a saturated solution of constituent ions in the presence of a seed crystal [13,14]. The absence of micro-crystals in the blood of any of the 63 patients with aortic stenosis, in our study, suggests that these crystals are initially formed in the valvular tissue and once formed are not released in the circulation. However, the positive association between serum calcium phosphate and aortic stenosis in our study, supported by others [7–9], leaves open the possibility that extra calcium and phosphorus in circulation may potentiate the growth of the mineral, once established in the valvular tissue. Pharmacological methods for reducing serum calcium or phosphate or both may therefore have a role in preventing the progression, if not the initiation, of the disorder.

Circulating inhibitors of calcification, may explain the lack of blood-borne micro-crystals. Fetuin-A, for example, is a liver-derived protein that is a potent circulating inhibitor of calcification [15]. It has been shown, in vitro, to inhibit spontaneous calcium phosphate hydroxyapatite formation from solutions supersaturated in calcium and phosphate [16] by rapid formation of soluble colloidal fetuin-A calcium phosphate complexes [17,18]. Such inhibitors may prevent precipitation of calcium phosphate even when serum levels are high. There is also a view that aortic valve calcification is not simply a physico-chemical process but an active one that involves in situ transition of valve interstitial cells to osteoblast-like bone forming cells [19] or transition of valve endothelial cells to mesenchymal cells with matrix effects giving rise to local calcium phosphate deposition or formation within the tissue itself [20].

It is possible that our method for detecting micro-crystals was insufficiently sensitive, with a lower detectable concentration limit of 0.008mg/mL. The synthetic calcium phosphate micro-crystals which we used in the validation study had similar size (about 200nm) to the calcium phosphate particles previously identified on valvular tissue [3] and these were easily identified, so we believe it is likely that scanning electron microscopy, which has a lower limit of resolution of about 1nm, would have detected crystals in the blood had they been present. Our method of sample preparation was tailored to detecting the calcified particles previously identified on aortic valve tissue, rather than calciprotein particles which exist as colloid in solution and require much stronger and longer separation methods. [21]

## Conclusion

Calcium phosphate crystals are a fundamental particle in the development of aortic valve stenosis and can be demonstrated using scanning electron microscopy. The results of our study indicate that in spite of higher levels of serum calcium and phosphate in patients with aortic stenosis, precipitation of calcium phosphate particles does not occur freely in the blood.

## Supporting information

S1 Fig Results of validation study showing concentrations of synthetic hydroxyapatite micro-crystals added to healthy volunteer blood samples and scanning electron microscopy images. Hydroxyapatite particles are indicated by the arrow. Scale bar = 10μm.
(EPS)
